# A Clinicopathological Report of a 93-Year-Old Former Street Boxer With Coexistence of Chronic Traumatic Encephalopathy, Alzheimer's Disease, Dementia With Lewy Bodies, and Hippocampal Sclerosis With TDP-43 Pathology

**DOI:** 10.3389/fneur.2020.00042

**Published:** 2020-02-12

**Authors:** Chunhui Yang, Sukriti Nag, Guoqiang Xing, Neelum T. Aggarwal, Julie A. Schneider

**Affiliations:** ^1^Rush Alzheimer Disease Center, Rush University Medical Center, Chicago, IL, United States; ^2^Department of Pathology (Neuropathology), Rush University Medical Center, Chicago, IL, United States; ^3^The Affiliated Hospital of the North Sichuan Medical College, Nanchong, China; ^4^Department of Neurological Sciences, Rush University Medical Center, Chicago, IL, United States

**Keywords:** Alzheimer's disease, chronic traumatic encephalopathy, dementia, dementia with Lewy bodies, phosphorylated tau, traumatic brain injury, TDP-43 pathology, tauopathy

## Abstract

Chronic traumatic encephalopathy (CTE) was recently recognized as a new tauopathy in which multifocal perivascular phosphorylated tau aggregates accumulate in neurons, astrocytes, and neurites at the depths of the cortical sulci. Traumatic brain injury (TBI) in early or mid-life is known to be associated with an increased risk of dementia in late life. This case report describes a 93-year-old former street boxer with a premortem diagnosis of severe dementia, who showed pathological evidence of the coexistence of Alzheimer's disease, CTE, dementia with Lewy bodies, and hippocampal sclerosis with TDP-43 pathology. These findings suggest that TBI may trigger a variety of misfolded proteins leading to dementia. Currently, clear clinical diagnostic criteria for CTE have not been established. Therefore, clinicians should be aware that TBI is a risk factor for dementia and that CTE can overlap with other neurodegenerative diseases.

## Introduction

Traumatic brain injury (TBI) was long recognized as a risk factor for dementia (1–5). Chronic traumatic encephalopathy (CTE) refers to the neuropathological changes resulting from repeated episodes of TBI (3, 5–7). In the early twentieth century, the terms “punch-drunk” or “dementia pugilistica” were used to describe the clinical features of a distinct neuropsychiatric syndrome that affected boxers. In 1949, the term CTE was used as synonymous with “punch-drunk” to describe the neurological deficits resulting from repeated blows to the head (8). Later, there were additional reports describing the neuropathologic features of this condition (9). In 2016, the first NINDS/NIBIB consensus meeting defined the neuropathological criteria of CTE and confirmed that the pathognomonic lesions in CTE are accumulations of abnormally hyperphosphorylated tau (p-tau) in astrocytes and neurons located around small blood vessels and at the depths of the cortical sulci (10). CTE, a new neurodegenerative tauopathy, was reported in athletes who played soccer, baseball, ice hockey, and rugby, as well as in military personnel exposed to explosive blasts (5–7, 11). Clinical presentation of CTE was divided into three phases: behavioral/psychiatric, cognitive, and motor (12, 13). Late/older onset cases present predominantly with cognitive impairment (14).

While CTE can be suspected clinically, at present, the definitive diagnosis of CTE can only be made following neuropathological examination of the brain (11, 15–17). CTE pathology contributes to the clinical presentation, and its interaction with comorbid neurodegenerative pathologies is unclear (9, 13, 18). This case report describes a late-life dementia case with a clinical history of TBI. Pathological examination of the brain showed CTE, with the coexistence of Alzheimer's disease (AD), dementia with Lewy bodies (DLB), and hippocampal sclerosis with TDP-43 pathology.

## Case Report

### Clinical Presentation

This patient was enrolled in the Rush Memory and Aging Project, a longitudinal study of aging and dementia, which was approved by the Institutional Review Board of Rush University Medical Center. A signed, informed consent was obtained for annual clinical evaluations and a signed Anatomical Gift Act for brain donation. The annual clinical evaluations were uniform and structured with a medical history questionnaire, neurologic examination, and detailed cognitive testing. Diagnosis of dementia followed a multi-step procedure as described previously (19, 20).

The deceased subject was a 93-year-old right-handed male, who was a street boxer in his 20s with no history of loss of consciousness. He first developed memory problems at the age of 82. Initially, he seemed more forgetful, although he was still able to live independently. However, at the age of 83, he started having difficulties in daily living, such as managing his own finances, managing his own calendar, and he became less social. He had trouble learning new information. He was not depressed; however, he was more irritable. His Mini-Mental State Exam (MMSE) score was 24/30. Magnetic resonance imaging of the head showed cortical atrophy, enlarged ventricles with mild enlargement of the cavum septum pellucidum. The clinical diagnosis was a major cognitive disorder due to AD. Although he was treated with cholinesterase inhibitors, there was rapid cognitive decline, and his MMSE dropped to 0/30 several years after the diagnosis of AD. There was no family history of dementia. He died from multi-organ failure at the age of 93.

## Methods

Autopsy was performed 8.5 h postmortem. The brain weight was 1,083 g with moderate, diffuse cortical atrophy. Blocks dissected from the brain included midfrontal, midtemporal, inferior parietal, occipital, anterior cingulate, and entorhinal cortices with amygdala, mid-hippocampus, basal ganglia (at the level of the anterior commissure), anterior thalamus, midbrain (at the level of the exiting third nerve fibers), and the cerebellum, which included the dentate nucleus. Blocks were processed using standard techniques, and paraffin-embedded sections (6 μm) stained with hematoxylin–eosin (HE) were used to detect microinfarcts and evaluate arteriolosclerosis and hippocampal sclerosis (HS) as described previously (21). A modified Bielschowsky stain was performed to demonstrate diffuse and neuritic plaques and neurofibrillary tangles (NFTs), which were quantitated in five brain regions (midfrontal, midtemporal, inferior parietal, and entorhinal cortices and hippocampus) that had the highest density of these structures as described previously (19, 20). Immunohistochemistry was performed to localize phosphorylated tau (p-tau; AT8), α-synuclein, β-amyloid, and phosphorylated transactive response DNA-binding protein 43 kDa (pTDP-43) using methods described previously (19, 21).

## Results

Microscopic examination (Table 1) showed.

**Table 1:** Summary of brain pathologies.

**Pathological diagnoses**	**Protein marker**	**Pathological changes**
**CTE—**primary features	p-Tau	Multifocal aggregates in a perivascular location and at depths of sulci
**CTE—**Supportive features	p-Tau	Severe changes in CA3, CA4, CA2, CA1, and subiculum of hippocampus
		Present in basal ganglia, amygdala, and substantia nigra
**Alzheimer's disease**		
Thal score 5	β-Amyloid	Based on distribution of β-amyloid plaques
Braak score 5	Tau	Based on neurofibrillary tangle scores
CERAD—probable AD	Tau	Based on neuritic plaque scores and history of dementia
NIA-Reagan—intermediate likelihood of AD	Tau	Based on CERAD and neurofibrillary tangle scores
**DLB, neocortical type**	α-Synuclein	LBs in substantia nigra, limbic sites, and neocortices
**TDP-43 pathology**	TDP-43	Present in amygdala, entorhinal cortex, hippocampus, and anterior temporal pole cortex

*CTE, chronic traumatic encephalopathy; p-tau, phosphorylated tau; CERAD, Consortium to establish a registry for Alzheimer's disease; DLB, dementia with Lewy bodies; TDP-43, transactive response DNA-binding protein 43 kDa*.

### CTE Tauopathy

**Figure 1:**
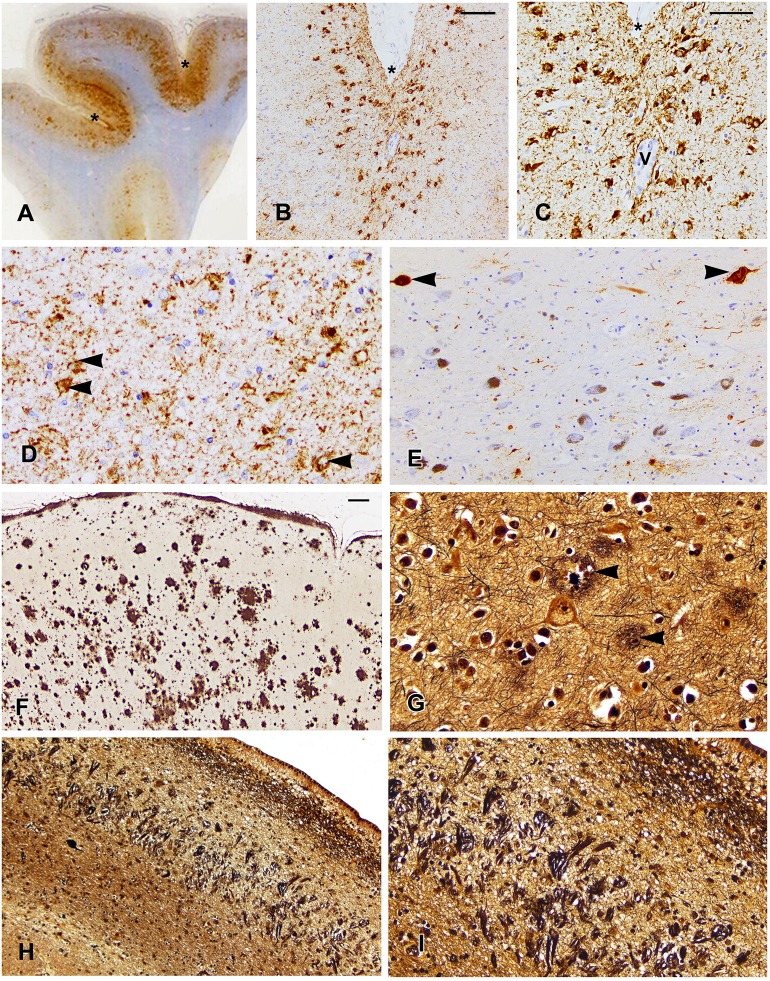
Localization of p-tau **(A–E)**, β-amyloid **(F)**, and tau in Bielschowsky-stained sections **(G–I)** are shown. **(A)** A whole mount of the parietal cortex shows increased p-tau immunoreactivity in the cortex at the depths of sulci, which are marked by asterisks in **(A–C)**. **(B)** Note the concentration of p-tau-positive neurons in the cortex at the depth of the sulcus **(B,C)** and surrounding a vessel marked by “V” **(C)**. **(D)** The CA1 sector of the hippocampus shows p-tau immunostaining in residual neurons (arrowheads) and in neurites. **(E)** The substantia nigra shows loss of neurons and tau immunostaining in two neurons (arrowheads). **(F)** Low magnification of the midfrontal cortex shows the extent of β-amyloid deposition. **(G)** The midfrontal cortex shows neuritic plaques (arrowheads). **(H)** A low power photomicrograph of the CA1 sector of the hippocampus shows dense collections of neurofibrillary tangles and ghost tangles, which are shown in higher magnification in **(I)**. Scale bars: **(B,F,H)** = 100 μm; **(C–E,G,I)** = 50 μm.

The NINDS/NIBIB Consensus criteria were used for the diagnosis and staging of CTE (10, 22). Findings required for the diagnosis of CTE were present and consisted of multiple perivascular foci of p-tau-positive aggregates in neurons, astrocytes, and neurites in all neocortical areas examined (bilateral middle frontal, middle temporal, and inferior parietal cortices). The p-tau accumulation was mainly concentrated at the sulcal depths (Figures 1A–C). In the hippocampus, all sectors showed p-tau pathology, which was particularly prominent in CA1 sector (Figure 1D). This pattern of NFTs in the hippocampus differs from that observed in AD. According to the Consensus criteria, the pattern of hippocampal involvement was consistent with the coexistence of CTE and AD (10, 18, 22). Abnormal p-tau immunoreactive neuronal and astrocytic aggregates were also present in the basal ganglia, amygdala, raphe nuclei, and substantia nigra (Figure 1E). The characteristic pathologic features of multiple foci of p-tau distribution throughout the neocortex, subcortical nucleus, brain stem, cerebellum, and hippocampus are consistent with a diagnosis of stage IV CTE.

### AD Pathology

β-Amyloid immunostaining showed widespread multifocal areas of Aβ deposits in the neocortex (Figure 1F), hippocampus, basal ganglia, cerebellum, and brainstem resulting in a Thal score of 5. In the Bielschowsky stained sections, quantitation of neuritic plaques (Figure 1G) along with the history of dementia provided a Consortium to establish a registry for Alzheimer's disease (CERAD) diagnosis of probable AD. Quantitation of NFTs showed a high density in the hippocampus (Figures 1H,I), which along with NFTs in the neocortical areas gave a Braak score of 5. A pathological diagnosis of high likelihood Alzheimer's disease was made using the NIA-Reagan diagnostic criteria (20). However, the unusually high density of NFTs predominantly in the CA1 of the hippocampus was consistent with a diagnosis of the coexistence of AD and CTE.

### DLB, Neocortical Type

**Figure 2:**
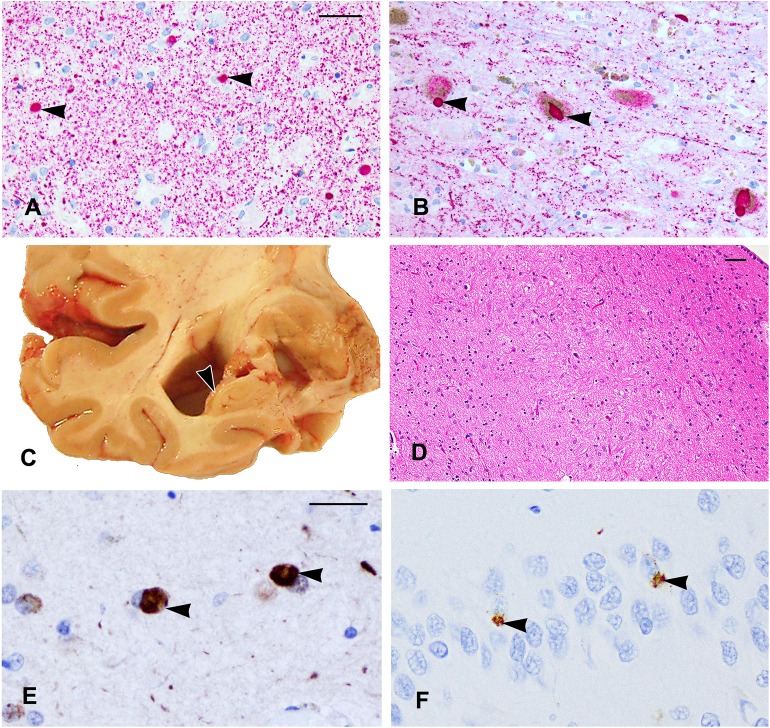
**(A,B)** α-Synuclein immunostaining shows Lewy bodies in the amygdala **(A)** and in the substantia nigra (arrowheads). **(C)** The right temporal lobe showing severe atrophy of the hippocampus (arrowhead) with enlargement of the adjacent inferior horn of the lateral ventricle. **(D)** A segment of the CA1 sector of the hippocampus shows few residual neurons and mainly glial nuclei due to hippocampal sclerosis. **(E,F)** TDP-43 cytoplasmic inclusions are shown in the amygdala **(E)** and neurons of the dentate gyrus **(F)** (arrowheads). Scale bars: **(A,B)** = 50 μm; **(D)** = 100 μm; **(E,F)** = 25 μm.

On α-synuclein immunostaining, LBs were identified in six different brain regions (midfrontal, mid-temporal, entorhinal and cingulate cortices, amygdala, and substantia nigral; Figures 2A,B). Lewy bodies in the substantia nigra were associated with mild neuronal loss and gliosis. Since LBs were present in the neocortex, substantia nigra, and limbic sites, this case met the pathologic criteria of DLB, neocortical type.

### Hippocampal Sclerosis With TDP-43 Pathology

Coronal slabs of the brain showed moderate enlargement of the frontal and temporal horns of the lateral ventricles associated with severe hippocampal atrophy (Figure 2C), and this was confirmed by microscopy of the HE-stained section, which showed severe neuronal loss and gliosis in the CA1 of the hippocampus compatible with a diagnosis of hippocampal sclerosis (Figure 2D). Cytoplasmic TDP-43-positive inclusions were found in the neurons and glia in the amygdala (Figure 2E), entorhinal cortex, CA1 sector, and the dentate neurons (Figure 2F) of the hippocampus and in the anterior temporal pole cortex. The degree of non-p-tau-related pathology in the hippocampus was greater than that described in the CTE cases (10).

## Discussion

This case report describes the clinical and pathological findings of a previous street boxer who developed severe dementia (MMSE = 0). Brain examination showed the coexistence of AD, CTE, DLB, neocortical type, and hippocampal sclerosis with TDP-43 pathology. The finding of five pathologies in a single case is rare.

Clinically, CTE is reported to have an early or late onset. The early/younger age onset subtype is characterized by behavior and mood symptoms but with minimal cognitive or motor impairment, while the late/older onset subtype is characterized by cognitive impairment (14). There is significant correlation between the pathological stage of CTE and the duration of TBI exposure and the age at death (22). Clinically, it is difficult to distinguish the CTE subtypes because comorbid pathologies are common in CTE and due to an overlap with symptoms of other neurological diseases (14, 16, 23). The subject in the present study had the late onset subtype of CTE. A recent report suggested that cognitive reserve may mitigate cognitive decline in older individuals with early life TBI (14). However, once cognitive decline occurs, cognitive deterioration is severe, possibly due to the contribution of comorbid pathology as in the present case. Dementia is reported to be associated with DLB, neocortical type, and the stage of CTE in addition to AD pathology and age at death (23). An early or mid-life TBI as a risk factor for dementia in late life is well-recognized (2, 3, 24).

There are reports of CTE cases with coexisting additional pathologies. In a group of 68 cases with a pathological diagnosis of CTE and a history of antemortem TBI, 85% had coexisting TDP-43 pathology, 11% had coexisting Alzheimer's disease, 16% had coexisting Lewy body disease, while 6% had coexisting frontotemporal lobar degeneration (25). In a group of six football players with TBI and progressive cognitive impairment, postmortem examination showed that AD and TDP-43 often coexist with CTE (5). In another study (18), of eight soccer and rugby players with dementia and a pathological diagnosis of CTE cases, there was coexistence of AD pathology in seven cases, of TDP-43 pathology in six cases, and one case showed CTE associated with DLB pathology, while in five cases, there was coexistence of three pathologies (CTE, AD, and TDP-43). None of the reported cases showed the coexistence of five pathologies in a single case as observed in the present case. The coexistence of multiple pathologies suggests that TBI may initiate or accelerate multiple proteinopathies resulting in several different neurodegenerative processes (3, 5) resulting in dementia (2–5). One of the mechanisms that results in the development of dementia after TBI is AD-related β-amyloid and tau pathology (26). However, how TBI triggers multiple proteinopathies remains uncertain.

It is well-established that p-tau is a biomarker for CTE. Although there are different tauopathies, the pathognomonic lesion of CTE, consisting of multifocal, perivascular p-tau aggregates in neurons, astrocytes, and neurites at the depths of the cortical sulci is distinct and not found in the other tauopathies (10). However, the neuronal p-tau found in CTE does share a similar profile regarding isoform ratio and phosphorylation state as the tau in AD (10). Both CTE and AD share the characteristic feature of hyperphosphorylated tau, where phosphorylation at specific residues occurs as an ordered process, leading to tau aggregation and oligomer formation (6). However, in the present case, the severity of CA1-predominant neurofibrillary degeneration in the hippocampus supports the coexistence of AD and CTE (10, 22). Nevertheless, the differential diagnosis of hippocampal p-tau pathology in CTE and AD requires further study (10). Aβ plaques, especially diffuse Aβ plaques, are present in some cases of CTE, but they are not a consistent feature of CTE and represent the coexistence of AD (10). Aβ deposition is reported to occur at an earlier age and at an accelerated rate and is associated with increased clinical and pathological severity in CTE (23).

Investigation of the clinical and pathological relationships between CTE and DLB in a group of deceased athletes reported that CTE significantly increased the odds of having DLB, neocortical type (23). A study based on community-dwelling older persons confirmed that DLB, neocortical type plays an independent role in cognitive impairment and has a deleterious effect on many aspects of cognition in older persons (27, 28). DLB, neocortical type provides an adequate explanation for cognitive impairment or dementia (27, 28), and it lowers both the level of cognitive function and increases the pace of cognitive dysfunction in persons with AD pathology. The additional effect of LB pathology appears to be highly deleterious, lowering global cognition by a full standard deviation while increasing the odds of dementia by 43-fold (28).

The association of TDP-43 pathology with hippocampal sclerosis is well-documented, and hippocampal sclerosis is reported to be more common in those aged >90 years (29). The distribution of TDP-43 pathology in the present case is greater than that described in CTE (10). TDP-43 pathology was reported in nearly half of the older community-dwelling persons (30). A previous study reported that TDP-43 pathology starts from the amygdala before spreading to the hippocampus and other brain regions in aging and AD (21). In the present case, since TDP-43 pathology extended to the anterior temporal pole cortex, this pathology could contribute to dementia since extension of TDP-43 pathology to the anterior pole cortex was reported to be associated with increased odds of dementia and impaired episodic memory (21). Contributing to dementia in this case is the finding of hippocampal sclerosis, which, with coexisting TDP-43 pathology, is associated with lower function in multiple cognitive domains (29). Further study is necessary to clarify the combined roles of CTE and hippocampal sclerosis with TDP-43 pathology in dementia.

Currently, an estimated 1.6–3.8 million concussions occur annually in the US, with American football, hockey, soccer, and lacrosse accounting for most of the sports-related concussions (31). Head impact in contact sports or in warfare that do not result in apparent clinical symptoms may still result in neuronal injury and late onset CTE and other neurodegenerative diseases including DLB and AD (9, 32). Additional research is necessary to determine the contribution of p-tau and other pathologies to the development of the clinical symptoms of CTE. Early diagnosis and effective intervention could be a key strategy to prevent TBI-induced CTE and the associated neurodegenerative diseases that lead to loss of brain function and cognitive deficits.

## Data Availability Statement

All datasets generated for this study are included in the article.

## Ethics Statement

The studies involving human participants were reviewed and approved by Rush University Medical Center. The patients/participants provided their written informed consent to participate in this study. Written informed consent was obtained from the individual(s) for the publication of any potentially identifiable images or data included in this article.

## Author Contributions

All authors listed have made a substantial, direct and intellectual contribution to the work, and approved it for publication.

### Conflict of Interest

The authors declare that the research was conducted in the absence of any commercial or financial relationships that could be construed as a potential conflict of interest.
